# Atypical haemolytic uraemic syndrome associated with a *CD46* mutation triggered by *Shigella flexneri*

**DOI:** 10.1093/ckj/sfu032

**Published:** 2014-04-11

**Authors:** Vicky Brocklebank, Edwin K.S. Wong, Rick Fielding, Timothy H.J. Goodship, David Kavanagh

**Affiliations:** 1 Renal Services Centre, Freeman Hospital, Newcastle upon Tyne, UK; 2 The Institute of Genetic Medicine, Newcastle University, Newcastle upon Tyne, UK

**Keywords:** CD46, complement, haemolytic uraemic syndrome, shiga toxin, *Shigella*

## Abstract

We present a case of haemolytic uraemic syndrome (HUS) triggered by *Shigella flexneri*. Of the *Shigella* species, only *S. dysenteriae* type 1 is said to produce Shiga toxin and consequently cause HUS. Investigation of the complement system in this patient revealed a *CD46* mutation. In individuals with mutations in complement genes incomplete penetrance of atypical HUS (aHUS) is seen, suggesting that a trigger, such as infection, is required for disease to manifest. In an era of complement modulatory therapy for aHUS it is important to be alert to unusual presentations of diarrhoeal-associated disease.

## Introduction

Haemolytic uraemic syndrome (HUS) is characterized by the clinical triad of microangiopathic haemolytic anaemia, thrombocytopenia and acute kidney injury (AKI). The typical form is more common and is caused by enteric infection with Shiga-toxin (Stx)-producing bacteria [1]. The most frequent pathogens responsible are Stx-producing *Escherichia coli,* particularly serotype O157:H7 (more recently O104:H4 [2]) and *Shigella dysenteriae* type 1 [1].

The non-shiga-toxin-associated form of HUS or atypical HUS (aHUS) is, in the majority of individuals, associated with inherited and/or acquired defects in the complement system resulting in complement over-activation [3]. Increased understanding of the pathogenesis of disease has led to the successful introduction of the complement inhibitor eculizumab in the treatment of aHUS [4].

Although there are anecdotal reports of eculizumab use in Stx-associated HUS [5], the lack of evidence from a controlled trial leaves clinicians uncertain as to its efficacy and therefore it is not routinely used in Stx-HUS.

We report a case of HUS associated with *Shigella flexneri* diarrhoea where subsequent investigation revealed a *CD46* mutation.

## Case report

A 38-year-old woman was referred by her primary care physician following return from a visit to Holland where she had experienced an episode of AKI. She had attended the emergency department with a 2-day history of watery diarrhoea and vomiting.

She was found to have AKI with a serum creatinine of 663 μmol/L. Admission haemoglobin was 10.9 g/dL, and platelet count was 113 × 10^9^/L with schistocytes on blood film. The serum lactate dehydrogenase (LDH) was raised at 1346 u/L and coagulation profile was normal.

Her past medical history consisted of severe asthma with long-term steroid use. Her renal function was normal with a creatinine of 90 μmol/L 2 months previously.

Following volume resuscitation her renal function did not improve and the microangiopathic haemolytic anaemia worsened. She did not have neurological or any other extra-renal manifestations. Treatment with high-dose intravenous steroids and therapeutic plasma exchange was initiated, and she received twice-daily exchanges for a total of 10 days.

*Shigella flexneri* was isolated in both blood and stool cultures. Intravenous piperacillin-tazobactam was administered and a putative diagnosis of Stx-HUS was made. Blood samples taken prior to the commencement of therapeutic plasma exchange demonstrated that ADAMTS13 activity was 78% of normal and C3 [1.25 g/L (0.68–1.38)] and C4 [0.24 g/L (0.18–0.60)] levels were normal. Polymerase chain reaction (PCR) analyses for Stx1, Stx2 and STEC were negative. Serum creatinine peaked at 744 μmol/L and she did not require renal replacement therapy. When she was discharged 21 days after admission her serum creatinine was 140 μmol/L.

The patient was reviewed 17 days following discharge with a serum creatinine of 85 μmol/L, a urine protein:creatinine ratio of 32 mg/mmol, and a normal platelet count and LDH. Although there was no family history of renal disease, because *S. flexneri* does not usually cause Stx-HUS and the PCR for Stx had been negative, we investigated for aHUS.

**Fig. 1.**
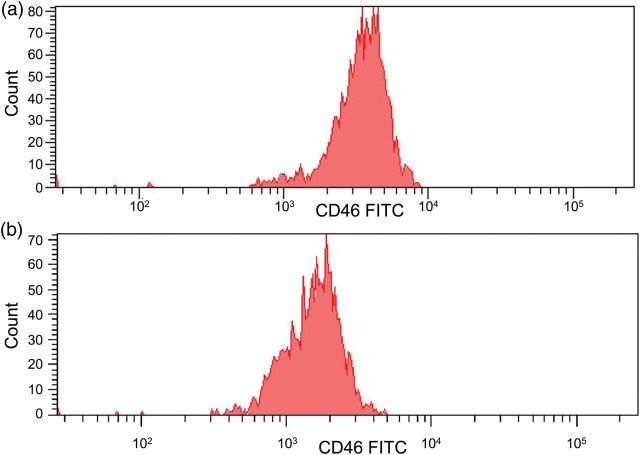
Flow cytometry analysis of CD46 expression on lymphocytes in a normal subject (**a**) and the patient (**b**) measured on the same day. FACS analysis was performed on the day of venepuncture. Mean fluorescence intensity for the patient was 1651 and 3658 for the control subject, consistent with haploinsufficiency of CD46.

Convalescent concentrations of complement components were C3 1.09 g/L (0.68–1.80), C4 0.17 g/L (018–0.60), factor H 0.73 g/L (0.35–0.59) and factor I 63 mg/L (38–58). Mutation screening of *CFH*, *CFI*, *CD46*, *C3* and *CFB* revealed a heterozygous *CD46* splice site mutation (IVS2+2T>G). This change has previously been identified in a patient with aHUS and demonstrated to result in abnormal splicing [6, 7]. FACS analysis of granulocytes from the patient demonstrated that cell surface expression of CD46 was reduced by 50% (Figure 1). In the following year she has had no further episodes of HUS, her serum creatinine is 88 μmol/L and urine protein:creatinine ratio is 6 mg/mmol.

## Discussion

This patient presented with diarrhoea and had clinical findings consistent with a renal thrombotic microangiopathy (not biopsy proven). A presumptive diagnosis of Stx-HUS secondary to *S. flexneri* infection was made and her renal function fully recovered. However, it is unexpected for *S. flexneri* infection to cause Stx-HUS. Of the *Shigella* species*,* only *S. dysenteriae* type 1 is said to produce the Shiga toxin responsible for HUS [8]. Although *S. dysenteriae* type 1 is the predominant cause of *Shigella*-associated HUS, a few cases of *S. flexneri* HUS have been reported [9]. The pathogenesis of non-*S. dysenteriae*-associated HUS is unclear. Recent studies have failed to detect shiga toxin sequences in non-*S. dysenteriae* type 1 [10] although historical reports suggest infrequent Stx-producing strains [11]. In this case the Stx-PCR was negative and we therefore re-evaluated the diagnosis and in particular investigated for atypical HUS.

Studies of familial aHUS have shown that not all individuals carrying a mutation in a complement gene will develop aHUS. It is now established that other factors, including a trigger, are often needed for aHUS to manifest [3]. In this patient we identified a functionally significant mutation (IVS2+2T>G) in the gene encoding the transmembrane complement regulator CD46. We presume that the *S. flexneri* diarrhoeal illness triggered aHUS in this patient. A preceding non*-E. coli* O157 diarrhoeal illness has been reported in 28% of patients with aHUS [12]. *Escherichia coli* O157 infections have also triggered the first presentation of aHUS in a patient with a CD46 mutation [13]. Another report described two patients with Stx-HUS resulting in end-stage renal disease (ESRD) who developed recurrent HUS following renal transplantation and were subsequently found to have complement gene mutations (*CFI*; p.V412M, *MCP* IVS2+2 T>G) [14].

The prognosis in individuals with aHUS associated with only a *CD46* mutation is good (0–6% risk of ESRD or death within 1 year of the first episode [12]). In keeping with this, our patient recovered normal renal function. In those individuals with a *CD46* mutation, who do progress to ESRD, the outcome following transplantation is better than patients with a mutation in *CFH*, *CFI*, *C3* and *CFB* [15]. This is because the renal allograft will usually correct the recipient's membrane-bound CD46 defect, whereas there is a high rate of recurrent disease post transplant in those individuals with serum complement protein defects.

This case highlights the importance of screening for underlying complement abnormalities in unusual presentations of diarrhoea-associated HUS. Making a diagnosis of aHUS in this patient is important because of the implications for her future management and for her relatives. She remains at risk (70–90% [12]) of recurrent episodes triggered by infection and so she has been advised to present whenever she is unwell so that appropriate investigations can be performed. In the event of a recurrence, therapeutic plasma exchange would not be expected to be beneficial because CD46 is cell surface bound and not a circulating protein [12, 15]. However, she would be a candidate for treatment with the complement inhibitor eculizumab, which is a monoclonal antibody directed against C5 [16]. In addition, her relatives have been counselled to present for assessment should they become unwell, although they have currently declined genetic screening.

Screening for underlying complement mutations is essential if an aHUS patient has progressed to ESRD and requires renal transplantation. This allows an individualized approach to the use of eculizumab to prevent the morbidity and mortality associated with recurrent aHUS in the graft [17].

In conclusion, HUS presenting in the context of a diarrhoeal infection should not be presumed to be Stx-associated. Non-Stx diarrhoea commonly triggers aHUS in individuals with underlying complement defects. Such patients may benefit from complement modulatory therapy.

## Conflict of interest statement

D.K. and T.H.J.G. have received honoraria for consultancy work from Alexion Pharmaceuticals. The results presented in this paper have not been published previously in whole or part, except in abstract format.
